# Conjunctival Lymphoma

**DOI:** 10.1038/s41433-022-02176-2

**Published:** 2022-07-26

**Authors:** Lindsay A. McGrath, David A. Ryan, Sunil K. Warrier, Sarah E. Coupland, William J. Glasson

**Affiliations:** 1 Queensland Ocular Oncology Service, Terrace Eye Centre, Brisbane, QLD Australia; 2 University of Queensland, School of Medicine, Brisbane, QLD Australia; 3 Sullivan Nicolaides Pathology, Brisbane, QLD Australia; 4 Liverpool Clinical Laboratories, Liverpool University Hospitals Foundation Trust, Liverpool, UK; 5 Department. of Molecular and Clinical Cancer Medicine, Institute of Systems, Molecular & Integrative Biology, University of Liverpool, Liverpool, UK

**Keywords:** Eye cancer, Lymphoma, Conjunctival diseases

## Abstract

Lymphoma of the conjunctiva is an ocular malignancy derived from clonal proliferation of lymphocytes. The majority of conjunctival lymphoma is extranodal marginal zone B-Cell lymphoma (EMZL), however diffuse large B-cell (DLBCL), follicular (FL), mantle cell (MCL) and T- cell subtypes are also seen. Clinical manifestations are non-specific, but include unilateral or bilateral painless salmon-pink conjunctival lesions. Approaches to treatment have centered around local immunomodulation, often with Interferon-α2b or Rituximab (anti-CD20 monoclonal antibody) with or without radiation. Although conjunctival lymphoma is generally considered an indolent disease, recent advances in next-generation sequencing have improved clinicians’ ability to predict future recurrence or systemic disease through assessment of cytogenic and molecular features. In this paper, we review the classification, clinical features, diagnostic techniques, and emerging strategies for management and prognostication of conjunctival lymphomas.

## Abstract (zh)

结膜淋巴瘤是一种源于淋巴细胞克隆性增殖的眼部恶性肿瘤。大多数的结膜淋巴瘤来源于淋巴结边缘区B淋巴细胞 (EMZL), 但也可见弥漫性大B细胞 (DLBCL)、滤泡细胞 (FL)、套细胞 (MCL)以及T细胞亚型。结膜淋巴瘤的临床表现无特异性, 但包括单侧或双侧无痛性浅粉色鱼肉样结膜病变。治疗方法通常以局部免疫调节治疗为主, 通常使用干扰素-α2β或利妥昔单抗 (抗CD20单克隆抗体)伴或不伴放疗。尽管结膜淋巴瘤通常被认为是一种惰性疾病, 但新一代测序的最新进展提高了医生通过细胞遗传学和分子特征预测未来复发率及全身疾病的能力。在本文中, 我们回顾了结膜淋巴瘤的分类、临床特征、诊断技术、治疗及预后的新兴策略。

## Background/Introduction

Lymphomas are malignant lymphoid tumours arising as clonal proliferations of either B-lymphocytes or T-lymphocytes, or less commonly natural killer (NK) cells. When occurring in the conjunctiva, they can be primary well-defined tumours, or secondary manifestations of systemic lymphoma. Although lymphoma in this region accounts for only 5–10% of all extranodal lymphomas, it is one of the most common adnexal malignancies [1, 2]. On clinical examination, it is not possible to differentiate between benign and malignant lymphoid lesions; therefore, tumour biopsy is essential to establish the diagnosis and prompt further systemic evaluation.

## Definition

The conjunctiva contains specialised lymphoid tissue, and acts as an antigen barrier as part of the mucosa-associated lymphoid tissue (MALT) system [3]. Antigen stimulation can lead to reactive lymphoid hyperplasia (RLH)—which usually contains equal proportions of polyclonal B and T- lymphocytes. B-cell type non-Hodgkin lymphomas (NHL) can also arise within the conjunctival MALT. Approximately 24 to 48% of lymphomas are extranodal – with the most common sites including stomach, tonsils or adenoids, skin and small intestine [2, 4]. Periocular lymphoma represents only 2% of all extranodal sites, with primary conjunctival lymphomas accounting for 25–30% of all lymphomas in this region [5]. The two low-grade B-cell NHL arising in the conjunctiva, include extranodal marginal B-cell lymphoma (EMZL) and follicular lymphoma (FL), while the two high-grade types are diffuse large B-cell lymphoma (DLBCL) and mantle cell lymphoma (MCL). EMZL is the most frequent lymphoma subtype found in the conjunctival MALT system (>50%) and is considered to arise from neoplastically transformed marginal zone cells in reactive follicles [1, 6].

Morphologically, the lymphoma cells infiltrate around reactive B-cell follicles and spread to form confluent areas of heterogeneous small B-cells (centrocyte-like cells, monocytoid cells and small mature lymphocytes) with scattered immunoblast- and centroblast-like cells [7]. Clinically, EMZL is usually characterised by an indolent course, and is considered a disease of the elderly.

## Historical aspects

Hochheim was the first to review the literature on the subject of conjunctival lymphoma, in 1900 [8, 9]. He reports the first known case of conjunctival lymphoma to be published by Arnold and Becker in 1872 [10]. These authors reported a single case of a bilateral conjunctival neoplasm, and speculated that the underlying disturbance may be congenital—but not ruling out the presence of lymphatic tissues in the conjunctiva. Hochheim reviewed 5 subsequent cases with histological correlation, and added a case of his own, a 79-year-old female with bilateral conjunctival lymphoma. In the 1890 s, Goldzieher and Greeff used the term ‘pseudotrachoma’ to describe conjunctival lymphomas, and this nomenclature was adopted by many subsequent authors at the time, due to the presence of large follicles and inflammatory symptoms [11, 12].

**Fig. 1:**
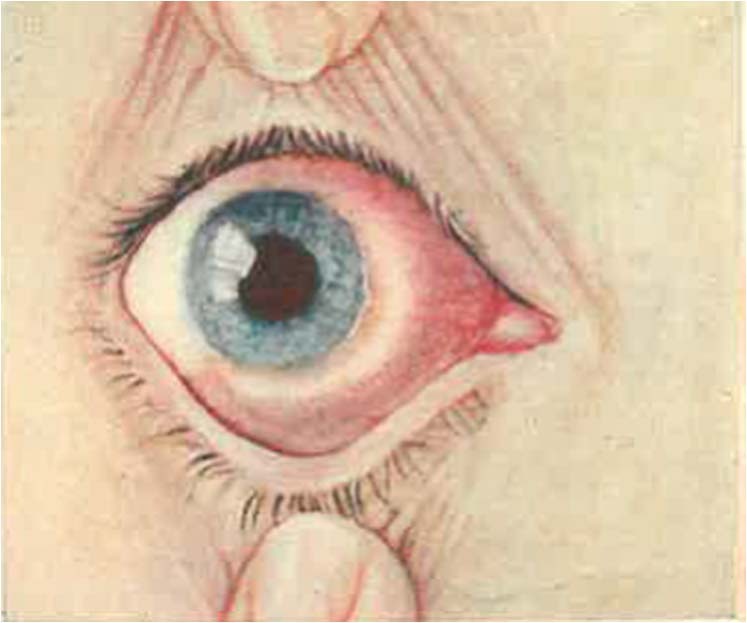
A case of “solid edema” (Lymphoma). Described by Bedell as a “rounded, elevated, peri-bulbar mass extending from the limbus backward fading into the surrounding tissue”. Reproduced with permission [14].

In his descriptive paper for the Lancet in 1929, Wright was the first to make the now ubiquitous comparison of the clinical colouring of the conjunctival lymphomas to a common fish: “..lymphomatous masses under the bulbar conjunctiva, rather resembling pieces of smoked salmon, which extended backwards into the orbit over the muscle insertions” when comparing his cases to those of Bedell—the first to describe conjunctival lymphoma in the USA (Fig. 1) [13, 14].

Friedenwald was one of the first, in 1929, to correctly hypothesise that lymphomas of the conjunctiva may occur with or without systemic disease[15]. Coats furthered this, by using the term ‘simple lymphoma’ to represent a purely local tumour reproducing accurately the histological character of lymphoid tissue and not associated with any constitutional disease. He thought that these lesions may arise from pre-existing lymphoid cells in the conjunctival fornix – a controversial stance for the time [16]. Much later, in 1952, Feinstein first reported on ocular involvement in systemic lymphomatous disease, drawing the conclusion that ocular or adnexal lymphoma should be regarded as a systemic disease, even if extraorbital spread does not manifest for several years [17].

In the decades that have passed since these initial reports, the concept of NHL arising from MALT has become a well-defined entity, after initial description by Isaacson and co-workers in 1984 [3]. More recently the World Health Organisation (WHO) classification of malignant lymphomas included marginal zone lymphoma of the MALT and listed several extranodal as well as nodal variants.

## Current WHO classification and general overview of lymphoma nomenclature

The WHO classification of Tumours of Haemopoetic organs was adopted from the Revised European American Classification of Lymphoid Neoplasms over 25 years ago [18]. The latest WHO classification revision published in 2017 comprises more than 80 entities of mature lymphoid neoplasms, which are defined according to their morphology, immunophenotype, genetic and molecular profiles, clinical features and cellular derivation [7]. Conjunctival lymphomas are predominantly extranodal NHLs, with the most common subtype being primary EMZL of MALT type (66%)[5, 19]. The second and third most common B-NHL are DLBCL (10%) and FL (8%), with rarer lymphoma subtypes—such as MCL and T-cell lymphomas—occurring very infrequently in this region [5, 19].

The most widely used staging system for malignant lymphomas is the Ann Arbor staging system, first adopted for Hodgkin disease in 1971 [20]. This system stages lymphoma with both nodal and extranodal disease, considering anatomic location and systemic symptoms. There are four stages (I-IV) according to lymph node group, location and organ involvement. The shortcomings of this system in the staging of MALT, and particularly ocular adnexal disease, were highlighted by Coupland and co-workers in 2009 [21]. Specifically—the disease extent of lymphomas in this site are not accounted for or possible to describe in detail using the Ann Arbor system, with most disease falling under Stage I-II (in particular IE, E for extranodal), regardless of location or size. The system does not discriminate between clinical or histomorphological high or low risk features – and fails to create accurate prognostic groups between patients.

The shortcomings in the application of the Ann Arbor system to ocular adnexal lymphoma (OAL) were addressed in the development of a TNM-based classification system of these tumours published in 2009 [21]. This user-friendly anatomic documentation of disease takes into account site-specific factors, and has created a common language for international collaboration. This was adopted in the American Joint Committee on Cancer (AJCC) Cancer Staging Manual 7^th^ Edition, and revised for the most recent 8^th^ Edition [22, 23].

These modern classifications stratify disease based on extent of regional nodal and distant systemic involvement at the time of diagnosis. Most conjunctival lymphomas (up to 90%) present as primary and well-circumscribed disease without evidence of prior or concurrent systemic disease [5, 24, 25]. Systemic dissemination is more frequent with high-grade subtypes, with up to 42% of patients with DLBCL and 89% of patients with MCL having evidence of systemic lymphoma at diagnosis[26]. Although rare, when T-cell lymphoma is encountered in the conjunctiva, up to 80% have a primary site elsewhere [24].

## Literature search

Pubmed (January 1964 to end of October 2021) and Medline (January 1946 to end of October 2021) databases were searched using the search terms conjunctival lymphoma and ocular lymphoma. Bibliographies were reviewed, and further papers were sourced dating back to 1872 for historic reference. Removing duplicate articles, excluding articles that were related to intraocular or orbital lymphoma, and removing most foreign language papers provided a total of 110 unique articles which were consulted and reviewed.

## Epidemiology

The worldwide incidence of OAL is approximately 0.2 per 100,000, and is seen in 1–2% of all NHL and 5–10% of all extranodal lymphomas [1, 2]. Conjunctival lymphoma ranks third behind squamous cell carcinoma and melanoma among the most common primary malignancies of the ocular surface [27, 28]. The age-adjusted incidence of conjunctival lymphoma is similar between European and North American populations [1, 29, 30]. The age-adjusted incidence of EMZL and FL in both Danish and American populations was seen to increase significantly during the period 1980–2005 across both genders, which parallels the rise in systemic NHL over a similar time period. Reasons for the observed increase in cases is unclear, but the authors hypothesise that an unidentified subclinical infectious agent, or increasing exposure to ultraviolet light may be implicated [1].

Kirkegaard and co-workers recently performed a major review of 1014 conjunctival lymphoid neoplasms, and 98% were found to be of B-cell lineage [24]. They found the four main subtypes to comprise: low-grade EMZL (81%); FL (8%); MCL (3%); and DLBCL (3%). These data were mirrored in a subsequent multicentre study of 263 OAL patients [24, 26].

Geographically, in a multicentre study, EMZL was found to be more common in the United States and India than the United Kingdom—potentially due to a selection bias. There was also a male predominance of EMZL and FL in India, consistent with previous reports [26].

## Special Populations

Current understanding of conjunctival lymphoma in paediatric populations (≤18 years) is limited to case reports and small case series’ [31–34]. In the largest series to date, Moustafa and co-workers reported retrospective series of 55 OAL in the paediatric population in 2020 [35]. The cases were collected from the United States National Cancer Institute’s Surveillance, Epidemiology and End Results (SEER) database, covering approximately 28% of the American population. As described in adults, Moustafa et al., found that EMZL represented the majority (46%) of cases in this population. Although the overall rates were still small, Burkitt Lymphoma (6%) and T-cell lymphoma (2%) were seen in higher percentages than in adults, while FL was less commonly observed [35]. Paediatric-type FL of the OAL has been reported in only 8 cases in the literature to date – exclusively in males [34, 36]. Overall, paediatric lymphomas in the ocular region are seen more often in the orbit than conjunctiva, and are more commonly seen in males, compared to females. The age-adjusted incidence rate for OAL was 0.12 per 1,000,000 [35].

In a report of benign and malignant conjunctival lesions in children, Shields and co-workers reported that lymphomas had a statistically significantly larger basal diameter, and locations of diffuse (rather than bulbar), superior (vs nasal), inferior (vs nasal) and diffuse (vs nasal) compared to RLH [37]. Thus, a lesion in the nasal bulbar conjunctiva is statistically more likely RLH, whereas in the superior or inferior conjunctiva is more likely lymphoma [37].

A single case report of the behaviour of conjunctival EMZL in a pregnant woman was published in 2017 [38]. There was no significant change to the lymphoma throughout pregnancy, and the patient was able to undergo treatment after delivery.

## Aetiology

Factors that predispose to conjunctival lymphoma have been of interest to researchers over the past 2 decades, and remain poorly understood. RLH is thought to be a potential precursor to lymphoma in some but not all cases, particularly in paediatric populations [7, 39]. Host immune deficiency, autoimmune conditions (Sjogren’s syndrome, Hashimoto’s thyroiditis, IgG4-related disease), genetic mutations and immune modulatory medications have been implicated in the development conjunctival lymphoma [40, 41]. Some chronic infectious agents, such as *Helicobacter pylori, Hepatitis C* and *Chlamydia spp*. have been proposed to play a role in the development of EMZL [42–44]; however, it would appear that there is distinct geographical variability between these organisms and the relationship to OAL, as well as differing detection methods [40]. The controversy regarding antibiotic use in OAL remains high given low incidence of Chlamydial or Helicobacter infections in this region [45, 46]. If *Chlamydia spp*. have been identified, it has been suggested that OAL patients with low tumour stage could be considered for antibiotic therapy as a first line treatment [46].

A recent study of conjunctival microbiota found evidence to suggest that *Delftia sp*. may play a pathophysiological role in the development of conjunctival EMZL, and *Bacteroides* and *Clostridium* may play protective roles [47]. Fluctuations in these bacterial compositions may cause disturbances of innate immunity. As above, the association between infectious agents and conjunctival lymphoma remains controversial and, if present, appears to vary by ethnical group and geographical region [45, 46, 48].

## Clinical features

### Ocular

The conjunctiva of the eye contains organised lymphoid tissue that plays a key role in the protection of the ocular surface by initiating and regulating immune responses [3]. This MALT can be easily identified as follicles on slit lamp examination. Although it does not seem to be present at birth, it increases to an average of 30 follicles / eye by age 10, and subsequently declines with age [49]. The tissue contains IgA, IgD and IgM expressing CD20+ B cells, macrophages, CD21+ follicular dendritic cells, as well as some CD3+ T cells in the surrounding areas [1, 49]. These cells are responsible for antigen presentation, activation, proliferation and cellular migration in response to microorganisms and antigenic substances. RLH can occur as a result of antigen stimulation of the MALT tissue. It is more common in younger populations, and histological studies show equal population sizes of polyclonal B- and T- lymphocytes[6]. The neoplastic counterpart of the marginal zone cells in these reactive follicles is termed EMZL. Lymphoma cells infiltrate around reactive B-cell follicles and spread to form confluent areas of heterogeneous small B cells[1].

In a study of 117 cases of conjunctival lymphoid tumours, Shields and co-workers reported that 85% were symptomatic at presentation [25]. The majority of these (67%) had minor complaints of a lump, irritation or eyelid ptosis [25]. Other possible symptoms include chemosis, hyperaemia, dryness, discharge, epiphora, ectropion, pterygium, symblepharon or photophobia [24].

**Fig. 2:**
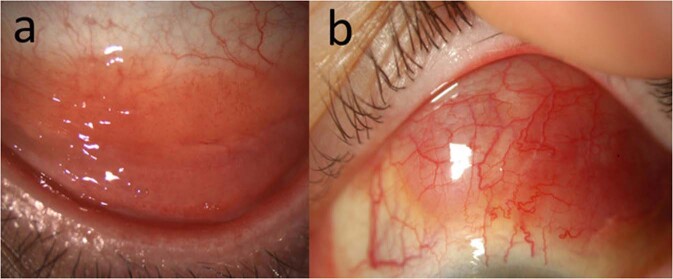
Clinical images of EMZL. **a** Inferior fornix with visible intrinsic vessels **b** superior bulbar locations.

Shields and colleagues saw unilateral lesions in 2/3 of presentations, which was similarly reflected in Kirkegaard and co-workers’ multicentre review reporting a unilateral manifestation in EMZL of 82% [25, 26]. Most tumours are seen to occur in the superior or inferior conjunctiva (62%), with most hidden under the lids or within the fornices, and only 7% reaching the limbus. The majority of conjunctival lymphomas (>90%) are pink in colour (with the remainder yellow or white) (Fig. 2a) [25]. The typical fleshy, “salmon patch” conjunctival swelling is similar in both RLH and lymphoma. Lesions are typically mobile and non-lobulated [48]. In approximately 1/3 of cases intrinsic vessels can be seen on slit lamp examination (Fig. 2b) [25]. Tumour base is 15 mm on average, with a thickness of 3 mm, and most patients have 2–3 tumours per eye [25]. Most conjunctival lymphomas are low grade B-NHL and indolent and do not show other concerning clinical features.

When differentiating between RLH or chronic conjunctivitis and lymphoma, Jung and co-authors reported that EMZL with follicular features were more often bilateral than those with a smoother “salmon patch” appearance [48]. Lymphomas with follicles also were not seen to respond to topical steroid therapy. Conjunctival EMZL is seen to differ in appearance in adolescence. Beykin and colleagues found that a ‘salmon-patch-like’ lesion in the plical area was more often RLH in this population [39]. In general, most conjunctival lymphomas in children present with a firm, elevated globoid mass [24, 50].

**Fig. 3:**
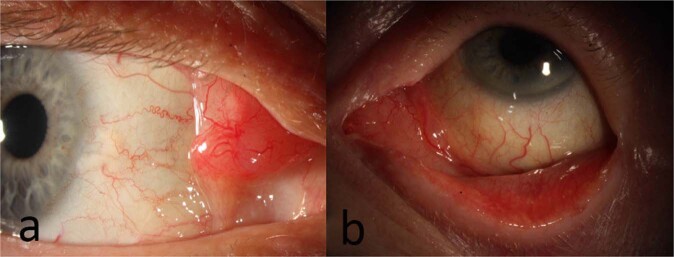
Follicular Lymphoma and DLBCL. **a** Slit lamp image of 69-year-old male with follicular lymphoma of the conjunctiva at the plica semilunaris with nodular appearance. **b** Slit lamp image of 81-year-old female with DLBCL of the conjunctiva involving the caruncle.

Less common lymphomas of the conjunctiva—DLBCL, FL and MCL—can present with irritation and a tumour mass [26]. In contrast to EMZL, FL is usually multinodular in appearance (Fig. 3a) [24, 36]. DLBCL tends to be more grey colour, rather than salmon pink (Fig. 3b) [51]. Lesions may show rapid growth, ulceration, invasion of surrounding tissues, feeder vessels and lymph node enlargement from regional spread [6]. Patients with MCL typically have larger lesions at presentation, with variable colouration, from salmon pink to dark red, and occur more commonly in elderly males [24]. SLL and conjunctival plasmacytomas tend to retain the typical non-lobulated “salmon-patch” appearance.

T-cell lymphomas of the conjunctiva are rare, but a high index of suspicion for these lesions can be held when there is concomitant episcleritis, scleritis or symblepharon at presentation [52]. Interestingly, up to 30% of T cell lymphomas will involve the limbus [24].

In a study of 63 patients for evaluation of the AJCC TNM criteria (7^th^ edition), Aronow and coworkers found that uveal involvement was present in 16% of patients with OAL. Conversely, in a series of patients with uveal lymphoma, 59% had involvement of ocular adnexal structures [53]. This continues to be a rarely recognised pattern elsewhere in the literature, however, highlights the importance of fundus examination in all patients with suspected OAL [54–56]. Patients with secondary uveal lymphoma (most commonly DLBCL) tend to have poorer visual acuity at presentation, bilateral disease, anterior segment involvement and clinically detectable vitreous cellular reaction/infiltration compared to patients with primary disease [57, 58]. Secondary uveal involvement can affect staging, and most importantly, needs to be considered during treatment. Involvement of the uvea is not specified in the 8^th^ edition of the AJCC, but there have been calls to include it in future revisions for these reasons [59].

### Systemic

The frequency of systemic involvement arising from a conjunctival lymphoma is rare but varies significantly with lymphoma subtype. About 2–5% of OAL occurs with concomitant systemic NHL. In contrast, high-grade DLBCL of the conjunctiva is related to systemic disease in 25–50% of cases, and up to 50–90% of cases of MCL are secondary [24, 26]. T-cell lymphoma is also usually a secondary disease (in 80% cases) [24].

Up to 20% of patients with primary conjunctival lymphoma will develop systemic disease, although this may be months, or even years later [25, 60]. Clinical clues to the presence or future risk of systemic lymphoma in patients with conjunctival disease include location of the tumour (fornix or mid-bulbar conjunctiva higher risk) and increasing number of discrete tumours [25].

There have been 2 reports of conjunctival EMZL spreading down the nasal cavity to the lacrimal sac [61, 62]. This is an important consideration and indication for imaging, particularly in patients who report nasal congestion, difficulty breathing or epiphora.

## Differential diagnosis

The differential diagnosis of conjunctival lymphoma (from most to least common) includes: chronic conjunctivitis, RLH, atypical pterygium, episcleritis, pyogenic granuloma, conjunctival amyloidosis, amelanotic melanoma, and, rarely, squamous cell carcinoma.

## Diagnostic techniques

The gold standard of conjunctival lymphoma diagnosis is an incisional biopsy for histopathological and cytological examination. Further ancillary studies are usually performed by the pathologist, including immunoprofiling via immunohistochemistry or flow cytometry and/or molecular studies. Prior to this, however, a complete ophthalmologic examination is important, as well as systemic examination. If a tumour is suspected, local and systemic imaging should be performed, as well as serological studies, and biopsy (Table 1).

**Table 1:**
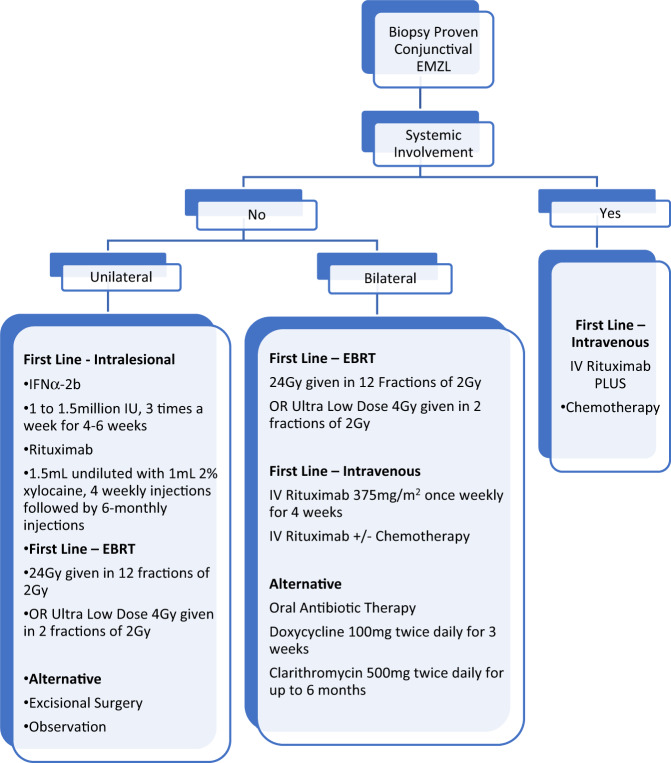
Investigations for suspected conjunctival lymphoma.

### Imaging

In patients with conjunctival MALT, complete imaging work-up is done for systemic staging. Cross sectional imaging with conventional modalities (Computerised tomography (CT) or magnetic resonance imaging (MRI)) of the orbits is often performed to evaluate retrobulbar disease and laterality. Interestingly, studies have shown that clinical examination by an ophthalmologist is superior to MRI for diagnosis of conjunctival MALT, with Nasser and co-workers showing only 18% of cases had MRI enhancement in the presence of disease [63, 64]. Imaging is advocated if there is uncertainty whether a lesion is purely conjunctival, because orbital lesions have a higher incidence of associated systemic disease.

**Fig. 4:**
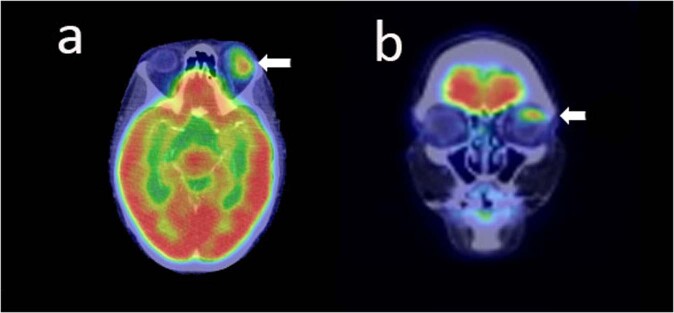
PET imaging in MALT. **a** Axial and **b** Coronal, ^18^F-FDG PET scan showing avidity at the left superior fornix, later confirmed to be EMZL.

In recent years, the emergence of advanced hybrid imaging techniques using positron emission tomography/CT (PET/CT) and PET/MRI systems have led to more accurate screening. PET is a diagnostic imaging technique that allows visualisation and quantification of biochemical processes via three-dimensional reconstruction of the bio-distribution of several positron-emitting isotope radiolabelled molecules. Hybrid PET/CT systems combine information from the PET component with the structural information obtained from CT allowing accurate anatomic localisation and morphological characterisation of lesions (Fig. 4a, b). Whole-body PET/CT post 18F-fluorodeoxyglucose (^18^F-FDG) administration has emerged as the standard of care for initial disease staging and post-treatment evaluation in MALT and orbital lymphoma. Even low-grade disease, these images are highly sensitive for accurate disease staging by detecting distant metastases missed on conventional imaging [65, 66].

English and Sullivan, in a series of 34 patients who underwent ^18^F-FDG PET at initial staging, demonstrated the enhanced performance of the modality in accurate disease staging, by detecting sites of systemic disease missed on CT, and therefore, significantly altering management strategies. However, this study also highlighted the limited performance of ^18^F-FDG PET in comparison to CT in detecting local OAL lesions, due to small lesion size and physiologic tracer uptake by extraocular muscles [67].

### Optical coherence tomography

High-resolution optical coherence tomography (HR-OCT) is a well-established imaging modality for the diagnosis and monitoring of retinal disease. Increasingly, anterior segment images obtained by HR-OCT allow clinicians to evaluate differences in tissue morphology and cellular patterns in various ocular surface conditions [68].

**Fig. 5:**
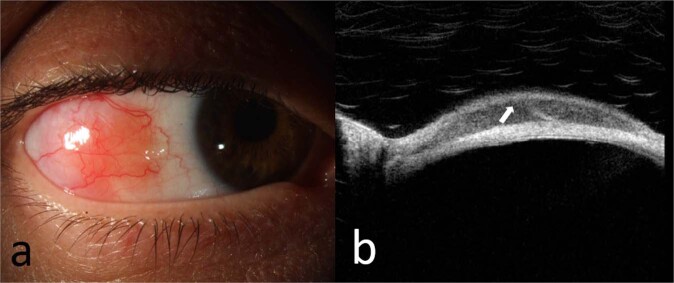
Ancillary imaging in conjunctival lymphoma. **a** Slit lamp photograph of a 46-year-old female with EMZL of the bulbar conjunctiva, **b** HD-OCT through the lesion showing normal epithelium with a hyporeflective, homogenous subepithelial infiltrate and a hyperreflective subepithelial band (arrow).

A recent study by Venkateswaran and co-workers described conjunctival lymphoma HR-OCT findings as homogenous, dark subepithelial lesions with smooth borders and monomorphic dot-like infiltrates (Fig. 5) [69]. These dots corresponded with lymphocytic infiltrates on histopathology. They also noted a subepithelial hyperreflective band. In contrast, confirmed cases of benign reactive lymphoid hyperplasia had more variability, and no hyperreflective band seen in paucicellular cases. Conjunctival amyloidosis cases appeared as heterogenous, subepithelial lesions with irregular borders and hyperreflective opacities [69]. Although HR-OCT is limited by shadowing in lesions of substantial thickness, it has been recognised as a useful tool in monitoring of disease resolution during treatment [70].

### Haematology

Initial staging for all extranodal lymphoma subtypes is often performed in conjunctival with a physician or haematologist. Serological testing is summarised in Table 1, and should include [71]:

- Full blood and differential counts,
- Biochemistry, including renal and liver function tests,
- Protein electrophoresis,
- Lactate Dehydrogenase (LDH) and β_2_ microglobulin (B2M),
- Serology for human immunodeficiency virus (HIV), Hepatitis B virus (HBV) and Hepatitis C virus (HCV).

Unilateral bone marrow aspirate (with morphology and flow cytometry) and biopsy is performed to confirm suspected stage I or II disease in all patients when local treatment is planned. Some limit these investigations to select patients with multifocal disease [71].

### Histopathologic examination

Formalin-fixed, paraffin embedded tissue sections are stained with haemotoxylin and eosin (H&E) to evaluate tumour morphology, and immunohistochemical (IHC) stains with antibodies directed against CD3, CD5, CD20 and CD79α are used to differentiate between T and B cell lineage. Additional IHC is used to further characterise the immunophenotype (Table 2). Monoclonality of the lymphoid cells can be identified with flow cytometry or using DNA extraction and examining for immunoglobulin heavy chain rearrangements using polymerase chain reaction (IgH-PCR) [5]. The tissues can also be examined using next generation sequencing to assess for cytogenetic and molecular features, including the mutational profile, which may be of value for the development of new treatments [72, 73].

**Table 2:** Immunoprofile of B-cell OAL.

	CD20	CD79a	CD3	CD5	CD10	BCL-6	BCL-2	CD43	Cyclin D1	CD21	CD23
EMZL	+	+	—	—	—	—	+	+ (25%)	—	+	—
FL	+	+	—	—	+	+	+	—	—	+	+/−
MCL	+	+	—	+	—	—	+	+	+	+	—
DLBCL-GCB	+	+	—	—	+	+	+/−	+/−	—	+/−	—
DLBCL-ABC	+	+	—	—	—	—	+/−	—	—	+/−	—

**Fig. 6:**
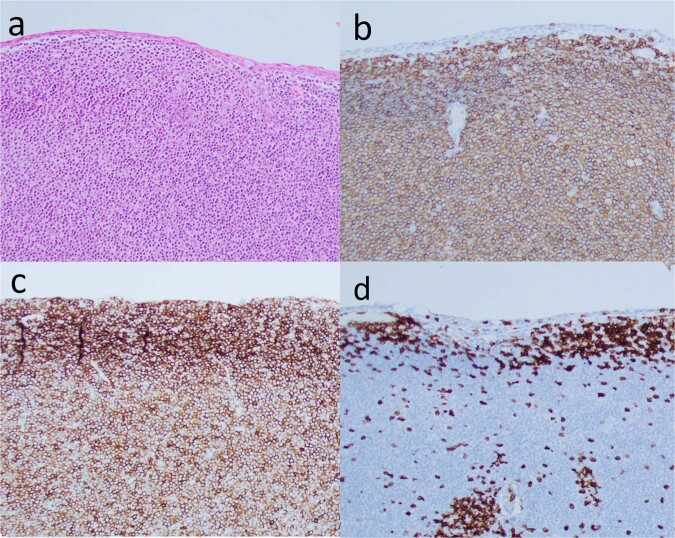
Histology of a conjunctival EMZL. **a** Haematoxylin and Eosin stain of tissue showing a lymphoid infiltrate within the substantia propria. **b** CD20 IHC shows predominance of positive B cells; **c** CD43 IHC demonstrates an aberrant co-expression by the neoplastic B cells; **d** CD3 IHC stain shows a background of small reactive T cells.

In conjunctival EMZL, there is an expansion of the marginal zone, and the substantia propria contains a diffuse monomorphic infiltrate of well differentiated heterogeneous lymphocytes (small B cells, cleaved centrocyte-like cells, monocytoid cells, small lymphocytes and scattered immunoblasts). Monocytoid cells with abundant pale cytoplasm are observed less frequently in EMZL of the conjunctiva, than other sites [74]. Plasmacytoid differentiation is frequent in EMZL [75]. The immunoprofile of EMZL is summarised in Table 2 and the histological and IHC features are demonstrated in Fig. 6. As expected in indolent tumours, there are usually few mitoses. Solid or sheet-like proliferations of large cells (usually BCL6+, CD10+) with high Ki-67 proliferation rates may herald higher-grade transformation to DLBCL.

The main differential of conjunctival MALT lymphoma, i.e., RLH, presents histologically as a polymorphous infiltrate composed of mature well-differentiated lymphocytes, often admixed with plasma cells that contain benign reactive lymphoid follicles or germinal centres. The reactive lymphoid follicles and the surrounding mantle zone contain CD20+ B lymphocytes, and CD3+ T cells predominate in the interfollicular zone. A monoclonal B cell population can be excluded by IHC, flow cytometry or IgH-PCR.

DLBCL may develop *de novo*, or may be the result of transformation from a less aggressive subtype, most commonly FL. They show diffuse effacement of normal histological architecture and are composed of large non-cohesive cells. There are large vesicular nuclei with multiple irregular eosinophilic nucleoli. CD20 is an important B cell marker, however this can be negative after treatment with Rituximab, and in such cases CD79a or PAX5 should be employed. DLBCL can be divided into at least three immunophenotypic subgroups, which correlate with the molecular subtypes – germinal centre (GCB), activated B-cell (ABC), primary mediastinal or those unable to be classified (up to 30%) [76].

Follicular lymphoma typically demonstrates densely packed multinodular follicles that lack tingible body macrophages. The follicular population (centrocytes, blastic centrocytes, centroblasts and immunoblasts) are uniformly atypical. In general, the prognosis worsens with greater proportion of centroblasts (per high power field).

MCL has a cytological similarity to EMZL, with a monomorphous B cell population closely resembling centrocytes. Aberrant CD5 co-expression by the B cells and cyclin D1 nuclear staining is important to differentiate these tumours from EMZL. The high frequency of systemic disease in these patients is also atypical for EMZL.

#### T cell lymphoma

Non-B cell lymphomas, derived from natural killer cells or T lymphocytes are uncommon and varied [7]. They differ in histopathological analysis, tumour site and cell of origin, and when affecting the ocular adnexa may represent an extension of mycosis fungoides affecting the eyelid or a secondary manifestation of peripheral T cell lymphoma not otherwise specified, anaplastic large T-cell lymphoma and T/natural killer lymphoma of the nasal type [52]. IHC evaluation for T-cell markers as well as T-cell receptor gene rearrangement studies substantiates T-cell neoplasia. Pan-T cell markers (CD2, CD3, CD5, and CD7) are usually expressed in variable degrees. Other markers that may be useful are anaplastic lymphoma kinase (ALK), TIA, Granzyme B, CD4, CD8, CD30 and CD56 [1].

A useful adjunct modality for demonstration of monoclonality and immunophenotype is flow cytometry. This testing requires fresh tissue. Although IHC demonstration of light-chain (κ and λ) restriction using paraffin embedded tissue can be used, it is not always reliable - as detection of cytoplasmic light chains in nonplasmacytic proliferations is prone to false-negative testing as light chains are labile and can be destroyed during routine tissue processing. Fresh tissue may also be useful for microbiological analysis, biochemical analysis and/or gene expression profiling.

Morphology and immunophenotyping described above remain the gold standard in diagnosis of conjunctival lymphoma, although there is an increasing repertoire of molecular tests available to enable future targeted therapies.

### Molecular pathology

The molecular processes involved in EMZL and DLBCL development are complex and our knowledge thereof is constantly evolving as we apply improved molecular analyses. The processes include chromosomal translocations, mutations caused by aberrant somatic hypermutation, sporadic somatic mutations, and copy number alterations, characterised by deletions and amplifications [7]. EMZL have similar clinical, morphological and IHC features between differing anatomical sites; however, recent evidence suggests that molecular genetic characteristics vary between the sites of origin [77].

Most recurrent chromosomal aberrations affect or target genes encoding for regulators of NK-κB. This gene is activated by the master transcription factor NF-κB which mediates lymphocyte development, activation and survival for regulated immune responses. Genomic profiling has also shown that in ocular adnexal EMZL, the A20 gene (NK-κB inhibitor) is inactivated by somatic 6q23.3 deletion and/or mutation in up to 30% of cases [78, 79]. This A20 (*TNFAIP3*) gene inactivation has been shown to be associated with poor lymphoma-free survival [76]. Toll-like receptor signalling can be linked to NF-κB activation with mutations in MYD88, which is seen in up to 7% of conjunctival EMZL, also conferring a poor prognosis [80]. Other known chromosomal alterations in ocular EMZL with respective frequencies include t(14;18)(q32;q21) 0–38%, t(11;18)(q21;q21) 0–15%, t(3;14)(p14.1;q32) 0–14% [76, 78]. These translocations target MALT1, BCL10 or FOXP1 genes, with incidences likely linked to specific premorbid geographical, environmental, embryologic conditions influencing transformation. Trisomy of chromosomes 3, 12 and 18 are frequent in all EMZL sites, but gain of chromosome 6 is more specific for the ocular adnexal region [79].

Multiple genetic aberrations are required for the development of FL, but the genetic hallmark has been recognised as t [14, 18] (q32;q21) translocation, involving BCL2 gene rearrangement. Ocular adnexal FL show this translocation in 76% of cases, with 4 to 6 additional genomic alterations seen on average [81]. The clinical heterogeneity of the disease is thought to be due to the multiple pathways involved in the lymphomagenesis of this subtype.

DLBCL prognosis is affected by MYC protein alterations, which are seen in up to 50% of these tumours [5]. This rearrangement can also be associated with BCL2 and/or BCL6 translocation—with such ‘double-’ or ‘triple-hit’ lymphomas considered high grade in the WHO classification [7]. Molecular or IHC testing should be carried out on DLBCL to determine ABC- or GCB subtype. In cases where these subtypes are encountered, additional testing for MYC and BCL2 IHC is warranted in order to determine prognosis [80]. Epstein-Barr virus (EBV) positivity has also been associated with a worse prognosis in patients over 50.

## Treatment

**Fig. 7:**
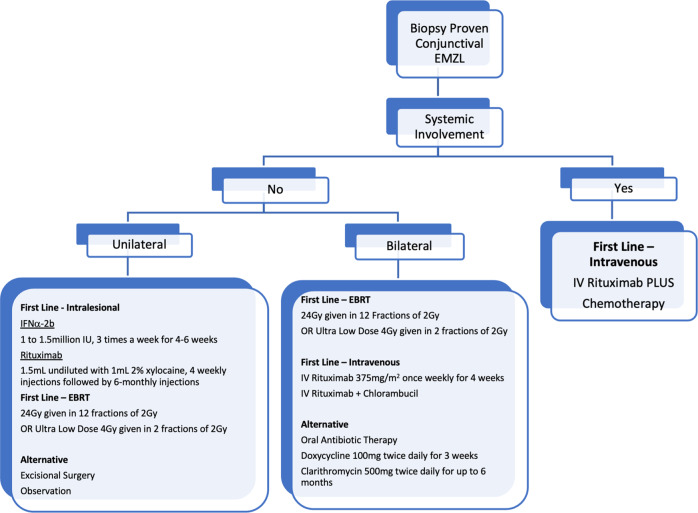
Approach to treatment of conjunctival lymphoma.

The management of conjunctival lymphoma is varied and tailored to the type of lymphoma, degree of dissemination and patient-specific factors. Management options include regional radiotherapy, chemotherapy and targeted therapies (Fig. 7).

In some cases, excisional biopsy or surgical resection may serve as therapy for well circumscribed conjunctival EMZL, however, in most cases attempts for complete resection should not be made because this does not influence survival and can lead to recurrence [82]. In exceptional cases where there are no signs of systemic lymphoma and the mass is small, Shields and co-workers have described techniques for excision. Given diseased tissue is localised to the conjunctival stroma, little loss of conjunctival epithelium is anticipated when meticulous dissection is employed, and adjuvant cryotherapy can be administered [25].

A “wait and watch” strategy has been proposed for elderly or frail patients with little symptoms, particularly in the setting of unilateral disease [83]. Treatment is recommended however, as progression of ophthalmic disease can occur, as can appearance of systemic disease [70, 82].

### Radiotherapy

Radiotherapy is the gold standard treatment for lymphoma isolated to the conjunctiva, classified as Ann Arbor stage 1, or T1-T2 (N0M0) according to the AJCC criteria. In a recent large multicentre study, stage 1E patients treated with EBRT monotherapy had a better survival than those treated with chemotherapy [84]. External beam radiation therapy (EBRT) has shown five-year local control rates of 89–100% in the setting of low-grade isolated OAL. The clinical target volume should include the entire conjunctival surface, including bulbar, forniceal and palpebral zones, while minimising treatment to adjacent lacrimal gland. A dose range of 20–30 Gy was long advocated for management of these lesions, however recent literature suggests much lower doses can be used [85, 86].

‘Ultra-low dose’ (or “boom-boom”) radiation therapy has been used for low-grade systemic lymphomas in recent years. Fasola and colleagues were the first to apply this to OALs in 2013, whereby patients were administered 4 Gy delivered in two 2-Gy fractions on two consecutive days [87]. In a study of 27 sites in 20 patients, they reported complete response in 85%, with only one patient (4%) requiring full dose EBRT [87]. Pinnix and coworkers published further evidence on the utility ultra-low dose EBRT for OAL in 2017 [86]. Patients with EMZL, CL or MCL had an overall response rate of 100% resolution of disease (86% complete, 14% partial) after a median 3.73 months following treatment [86]. Long term control was reasonably well maintained at 75% after two years.

Despite these promising findings, there is recent level 1 evidence demonstrating superior long-term control with 24 Gy EBRT over ultra-low-dose 4 Gy EBRT in extraorbital sites [88]. Jackson and coauthors recently showed that single-agent rituximab and ultra-low-dose EBRT had excellent local and systemic disease control with minimal toxicity, and this combination treatment could be considered in the future for conjunctival or orbital disease [89].

Although there are few complications with ultra-low dose EBRT, early minor complications of low dose EBRT include eyelid irritation and mild conjunctivitis. Long-term complications, which occur in up to 50% of patients with doses greater than 20 Gy, include dry eye syndrome, cataract formation, retinopathy, orbital fat atrophy, and keratitis [90, 91].

Brachytherapy can deliver high doses of radiation to target tissues, while minimising collateral exposure to surrounding healthy tissues. The use of a bidirectional Strontium-90-yttrium-90 applicator was reported in a small series by Regueiro et al. [92]. Local control was achieved in 77%, however a large percentage of late complications occurred in over 50% of eyes, which has reduced the uptake of this radiation modality.

### Systemic therapy

In the setting of conjunctival disease with evidence of lymphoma at other sites, systemic chemotherapy is often indicated. The standard single-agent chemotherapy for non-Hodgkin’s lymphoma remains chlorambucil, whereas combination therapy used includes cyclophosphamide, vincristine and prednisone (CVP), and cyclophosphamide, hydroxydaunorubicin, vincristine and prednisone (CHOP) [24, 91]. Further, in patients with high grade localised disease, systemic chemotherapy may also be considered if there are contraindications to, intolerance or poor response to first line EBRT.

Rituximab, a recombinant antichimeric CD20 monoclonal antibody has been used as a first line agent for cases of indolent B cell lymphoma in the ocular adnexa. Nuckel and co-workers were the first to employ rituximab at an intravenous dose of 375 mg/m^2^ weekly (for 4 weeks) in 2 patients with orbital disease, with a 50% complete response rate [93]. Similarly high rates of inadequate response in periocular disease have been reported, probably as a result of the poor bioavailability of the antibody and its effectors in the tumour microenvironment, which have limited the first line use of this therapy in this setting [28, 94]. In contrast, Rituximab has been found to result in a markedly improved outcome in patients with more aggressive ocular adnexal DLBCL and MCL, or in those with stage IIIE/IVE EMZL [84]. Rituximab-chemotherapy combination therapy (e.g., Rituximab-Chlorambucil or Rituximab-CHOP (R-CHOP)) is associated with better outcomes in patients with FL, MCL and DLBCL compared to chemotherapy alone [24, 95]. In the largest randomised trial for MALT lymphoma, the IELSG-19 study, a three-arm protocol was used comparing 6 weeks continuous chlorambucil+/− Rituximab versus Rituximab-monotherapy [96]. There was significantly better survival at 5 years for combination therapy (68% vs 51%) and overall response rate (95% vs 86%). Overall survival was not significantly different, but nevertheless, Rituximab-chlorambucil should be recommended in EMZL patients requiring systemic therapy [96].

Proponents of first-line chemotherapy state that this treatment option eliminates local complications from radiotherapy [28]. Although there are no direct studies comparing treatments in the conjunctiva, the collective rate of systemic relapse for radiotherapy is 6.2%, which is less than that currently reported for chemotherapy (9.3%) [28].

### Local therapy

As seen in other organ-specific disease, local therapies are better tolerated than intravenous and oral chemotherapies, with the latter often associated with anaemia, neutropaenia, parasthesia, hepatotoxicity, constipation and hyperglycaemia. Given the relative indolence of localised conjunctival lymphoma, compared to its systemic counterpart, clinicians have sought local therapies with more favourable side effect profiles.

There are very few reports of local rituximab use in conjunctival EMZL. The largest series to date was published in 2020 by Demirci and coauthors [97]. After diagnosis, 50 mg/5 mL of Rituximab was injected in the subconjunctival space around the tumour, monthly. Complete response was note in 73% of patients and partial responses in 27% after a median of 2 injections. Although this response rate was lower than EBRT, the authors concluded that this treatment is unlikely to cause adverse effects, is cost effective and may eliminate the need for radiation and its associated morbidity. An alternative dosing was proposed by Ferrrari et al. who administered 1 to 2 mL of undiluted rituximab (10–20 mg/mL) plus 1 mL of 2% xylocaine [94]. Injections were administered 4 weekly, followed by 6-monthly dosing if required. There was excellent tolerability, and an overall response rate of 65% with 5-year progression free survival of 59%. Although most studies of intralesional therapy are in the more common EMZL, there are anecdotal reports of intralesional rituximab use for FL [98].

Human interferons are a multigene family of inducible cytokines capable of exerting antitumour effects. In addition to enhancing the adaptive antitumour immune response, they can increase the expression of the tumour suppressor gene p53, inhibit angiogenesis and prime apoptosis in tumour cells [99]. The major disadvantage of Interferon is the short half-life, necessitating frequent treatment administration.

Local immunotherapy with interferon-α2b is another treatment option for conjunctival EMZL, first described in 1996 [100]. Subconjunctival instillation of 1–1.5 million international units (IU) of Interferon in 0.25 mL is advocated—with treatment frequencies up to 3 times weekly for 4–6 weeks, with further cycles advocated in the presence of incomplete response [70, 91, 99]. Studies of this promising treatment modality show remarkable long-term effectiveness, with approximately 85% of patients being progression or recurrence free during a 5-year follow-up period [24]. Side effects include temporary conjunctival chemosis, transient flu-like symptoms, pain, burning, or subconjunctival haemorrhage.

### Antibiotic Therapy

Although antibiotic therapy has been established as the standard of care in patients with gastric MALT lymphoma, much less is known about the value of antibiotic therapy in extragastric MALT. Given the controversial link between EMZL and *Chlamydia psittaci*, the routine use of topical or systemic antibiotic therapy as a first line regimen is not recommended. Doxycycline 100 mg twice daily has been shown to be a viable treatment option for T1N0M0 lymphoma with a 5-year progression free survival of 55%, where *C. psittaci* was confirmed [101]. A review of published literature detailing antibiotic use in MALT lymphoma concluded that although treatment responses have been reported, the data are not yet mature enough to draw definite long-term conclusions [102]. A three-week course of oral Doxycycline may be considered in asymptomatic patients, or those with positive *C. psittac*i swabs, but there is no definitive evidence for routine use, particularly in geographical areas with low baseline infection rates.

Clarithromycin is a substrate of P-glycoprotein which can induce apoptotic changes in tumour cells, and has been shown to exhibit antitumour activity in different murine cancer models. There has been only one report to date describing 6 months of oral Clarithromycin (500 mg twice daily) in relapsed or refractory conjunctival EMZL [103]. Eleven patients with OAML were included in the trial. The response rate was 45% and seems comparable to studies using doxycycline, although more trials are required [102]. A further 8 patients were included in a review of extragastric EMZL which showed overall response rate of 48% across all anatomic locations [104]. Based on the favourable toxicity profile, there are increasing proponents for the use of this medication for antiproliferative treatment in patients with low tumour burden in the conjunctiva [104].

### Cryotherapy

Cryotherapy is an effective modality, which continues to see use in the management of primary acquired melanosis, conjunctival melanoma, conjunctival epithelial neoplasia and squamous cell carcinoma. The use of cryotherapy in conjunctival lymphoma is not well established. In cases of local resection, Shields and co-workers advocate the use of adjuvant cryotherapy [25]. A single series of 42 cases of conjunctival lymphoma treated with cryotherapy was published by Eichler and Fraunfelder in 1994 [105]. They found a 98% rate of tumour resolution after 1–3 treatments [105].

## Prognosis

The prognosis for conjunctival lymphoma is universally good, with an overall 5-year survival of up to 94%, depending on the histological subtype, TNM stage at diagnosis and patient age [70, 106]. The most important of these prognostic factors are histological subtype, with more favourable 5-year survival rates seen in EMZL (97%) and FL (82%) in a large multicentre trial [26]. Poor prognosis was specifically seen in patients with age older than 60 years (for EMZL), female sex (for FL), T category exceeding T1 (AJCC, for DLBCL) or stage IE (Ann Arbor (for EMZL and FL) [26].

In a recent retrospective review of 140 patients with OAL, the 8th edition AJCC criteria were validated, with the histological subtype, lymph node involvement (≥N1), extraorbital involvement (M1) confirmed as conferring increased risk of relapse, as well as tumour diameter >30 mm and Ki-67 index >10% [60].

It is well established that patients with elevated LDH or concurrent hepatitis C infection have also shown to have poor rates of progression-free survival [70]. The prognostic value of cell-cycle associated markers in disease-free survival and lymphoma related death has been investigated, and lymphoma associated transcription factor BCL-6, MUM1/IRF4 and MIB1/Ki-67 have been associated with a higher risk of disseminated disease. In addition, t(14;18)(q32;q21) translocation show a trend to shorter progression free survival [1].

Bilaterality continues to be debated as a risk for disseminated disease. A study of 117 patients by Shields and co-workers found that 17% of patients with unilateral conjunctival lymphoma had systemic lymphoma at the time of diagnosis, increasing to 47% in the context of bilateral conjunctival disease at presentation [25]. As expected, given lymphocytes preferentially disseminate through the lymphatic route, the most common sites of tumour distant from the conjunctiva were lymph nodes (53%), followed by abdomen (19%), bone marrow (8%), brain (6%) and lung (3%) [25]. They were also able to correlate clinical factors at first visit predictive of systemic lymphoma by univariate analysis – including location of extralimbal site (fornix or midbulbar conjunctiva) and increasing number of discrete tumours [25].

## Conclusion

Conjunctival lymphoma commonly affects elderly patients, although some subtypes, such as EMZL can occur in younger populations. Gender distribution varies according to lymphoma subtype, with EMZL and FL showing a female predominance. In most situations, conjunctival lymphoma is asymptomatic, but patients may present complaining of redness or ocular surface irritation. Histologic subtype and clinical stage of lymphoma are the best indicators of prognosis and patient outcome.

Each lymphoma subgroup has a unique clinical course and requires tailored management with a multidisciplinary team. Local treatment administration is favourable in low grade lymphomas, with chemotherapy and/or radiotherapy reserved for higher grade lymphomas. The pathogenesis of conjunctival lymphoma is still not fully understood, but with the rapid advances in molecular analysis and immunophenotypic, we expect treatment modalities to improve and be tailored to patient’s specific presentations – with further improvement in disease-free survival.
